# Incidence and prevalence of organ toxicities in patients suffering from clear cell renal carcinoma treated with sunitinib and its impact on survival: a reference cancer center experience

**DOI:** 10.3389/fonc.2025.1590163

**Published:** 2025-08-20

**Authors:** Agata Sałek-Zań, Mirosława Püsküllüoğlu, Justyna Jaworska, Agnieszka Pietruszka, Joanna Lompart, Marek Ziobro, Tomasz Banaś

**Affiliations:** ^1^ Department of Clinical Oncology, Maria Sklodowska-Curie National Research Institute of Oncology, Kraków, Poland; ^2^ Department of Radiation Oncology, Maria Sklodowska-Curie National Research Institute of Oncology, Kraków, Poland

**Keywords:** renal cell carcinoma, tyrosine kinase inhibitors, adverse events, survival, sunitynib

## Abstract

**Introduction:**

Tyrosine kinase inhibitors (TKIs) are the standard treatment options for advanced clear cell renal cell carcinoma (ccRCC), but their toxicities can hinder optimal dosing, affecting clinical outcomes.

**Material and methods:**

A retrospective analysis of 96 patients treated with first-line line sunitinib at the National Research Institute of Oncology, Branch Kraków, Poland was conducted to assess the incidence and prevalence of organ toxicities in ccRCC and their impact on overall survival (OS).

**Results:**

The study included 96 patients. The median number of treatment cycles was 11 (IQR: 19), and the median duration was 63 weeks (IQR: 95). The most common toxicities were gastrointestinal (76.0%), fatigue (61.5%), and cardiovascular (49.0%), with 81.3% of patients experiencing multi-organ toxicity. Dose delays occurred in 37 patients (38.5%), mainly due to gastrointestinal (38.5%) and cardiovascular toxicity (21.9%). Dose reductions were required in 64 patients (66.7%), primarily for gastrointestinal (39.6%) and cardiovascular (16.7%) complications. Cardiotoxicity (p=0.017) correlated with improved OS. No OS differences were observed in enterotoxicity, hematologic, endocrine, dermatologic, or renal toxicity. Patients requiring dose reduction due to cardiotoxicity (p=0.012), hematologic toxicity (p=0.004) or gastrointestinal toxicity (p=0.004) had better survival than those without modifications. Patients requiring dose reduction due to any cause had better OS than those maintaining the initial dose. The timing or frequency of dose reductions had no significant impact.

**Conclusions:**

Cardiotoxicity, gastrointestinal and hematologic toxicities requiring dose reduction were associated with improved survival, suggesting these toxicities may reflect treatment efficacy. The findings emphasize the need to balance toxicity and treatment continuity.

## 1 Introduction

Renal cell carcinoma (RCC) is a global health concern, with 434,419 new cases and 155,702 deaths in 2022. Its incidence is higher in regions with a high Human Development Index, driven by obesity, hypertension, and improved diagnostics (1). Clear cell RCC (ccRCC), comprising 80% of renal tumors, has unique genetic features (2, 3). While localized cases may be cured by resection, recurrence and metastasis are common, influenced by clinical and therapeutic factors (4). Advances in clear cell RCC (ccRCC) pathogenesis, particularly Von Hippel-Lindau (VHL) gene dysregulation, have led to vascular endothelial growth factor receptor (VEGFR) tyrosine kinase inhibitors (TKIs) such as sunitinib, pazopanib, and cabozantinib, which are treatment options for advanced ccRCC by targeting VEGF-driven angiogenesis (5–7). While improving progression-free survival (PFS) and overall survival (OS) in advanced ccRCC, these agents’ toxicities often limit treatment duration, affect quality of life (QoL), and require dose adjustments, impacting adherence and survival (6, 8–11). Therefore, understanding the incidence and prevalence of these toxicities is vital for optimizing treatment strategies and improving survival in ccRCC patients.

TKIs toxicities can reduce the ability to maintain optimal dosing, which is critical for maximizing clinical benefits in patients with advanced ccRCC. Gastrointestinal toxicities, including diarrhea, nausea, and hepatotoxicity frequently occur with TKIs (9, 12). Cardiovascular toxicities, notably hypertension and thromboembolic events, are common with TKIs. Sunitinib can cause grade ≥3 hypertension in 15%-49%, with hypertension proposed as a biomarker of VEGF inhibition efficacy (6, 13, 14). However, uncontrolled hypertension may necessitate dose reductions or treatment interruptions, potentially compromising therapeutic efficacy and overall survival. Furthermore, dermatologic toxicities, such as hand-foot syndrome, are common in patients receiving VEGFR-TKIs. Some data suggest that patients treated with sunitinib who develop skin toxicity may experience longer survival (15). Though rarely life-threatening, adverse events (AEs) impair QoL and may require treatment changes affecting disease control. Studies suggest toxicities like hypertension, neutropenia, hypothyroidism, and skin reactions may indicate effective VEGF pathway inhibition and better responses (13–17). Managing toxicities to ensure continuous treatment without compromising quality of life remains a key clinical challenge (6). Ultimately, the management of toxicities associated with VEGFR-TKIs in ccRCC is a balancing act that requires careful monitoring and proactive intervention. The increasing number of new cancer patients is being fueled by an aging population and advancements in diagnostic techniques. Innovative treatments have the potential to enhance patient outcomes, including lowering mortality rates among working-age individuals, which can help mitigate productivity losses (18).

The aim of the study is to evaluate type and prevalence of sunitynib toxicity during first-line therapy of clear cell renal cancer patients. Additionally we assessed the impact of toxicity on the treatment effect.

## 2 Materials and methods

### 2.1 Study cohort and data collection

A retrospective analysis included 96 patients treated with sunitinib at the Clinical Oncology Department of the Maria Skłodowska-Curie National Research Institute of Oncology, Branch in Kraków, from January 2019 to June 2022, due to advanced RCC in a clinical practice setting. Before starting treatment, patients signed informed consent for the proposed therapy.

The qualification criteria included: diagnosis of stage IV renal cell carcinoma as defined by the Union for International Cancer Control (19), favorable or intermediate prognosis according to the Memorial Sloan Kettering Cancer Centre (MSKCC) scale (20), the presence of at least one measurable lesion in imaging studies conducted before qualification for treatment and defined according to the Response Evaluation Criteria In Solid Tumors (RECIST) version 1.1 (21). Additionally, for the purposes of this analysis, patients were retrospectively assigned to specific prognostic groups according to the International Metastatic Renal Cell Carcinoma Database Consortium (IMDC) scale (22), Clinical and pathological data were obtained from medical records and were anonymized. Adverse events were assessed according to Common Terminology Criteria for Adverse Events v 5.0 (CTCAE v 5.0) (23).

In case of AEs Grade ≥3, in accordance with the applicable guidelines and recommendations contained in the summary of product characteristics, therapy should be discontinued (7, 24, 25). In the event of a reduction in the intensity or cessation of a particular adverse event, it was possible to return to therapy at the initial dose or at a reduced dose (12, 24, 26).

### 2.2 Treatment protocol

Patients were treated in accordance with the European Union-approved prescribing information for sunitinib. Supplementary Information contains additional details. Treatment was continued until disease progression, the occurrence of unacceptable toxicity, patient death or withdrawal of consent.

Sunitynib is medication administered orally. The dosing regimen for sunitynib is 50 mg per day for 28 days, followed by a 14-day break,. In the situation of adverse event, a reduction to 37 mg per day may be possible, followed by a reduction to 25 mg per day (24).

### 2.3 Ethical approval

The study received approval from the Bioethics Committee at the Maria Skłodowska-Curie National Research Institute of Oncology – Warsaw Branch (registry number 6/23 dated October 5,2023).

### 2.4 Statistical analysis

Clinical features, showing non-normal distributions (confirmed by the Kolmogorov–Smirnov test), are presented as medians with interquartile ranges (IQR) or as numbers with percentages (%). Comparisons were made using the Mann–Whitney U test or Kruskal–Wallis ANOVA, with *post hoc* tests where necessary. Spearman’s rank test assessed correlations. Kaplan–Meier survival curves were generated based on toxicity incidence and prevalence, and Cox’s proportional hazard model identified survival predictors. The F-Cox test compared survival across patient groups. Using Cox proportional hazard model Hazard Ratio (HR) and 95% confidence interval (CI) were calculated for all cases with significant differences in survival curves. A p-value <0.05 was considered significant. Analyses were performed using STATISTICA 12.0 (StatSoft, Tulsa, OK, USA) and MedCalc 17.0.4 (MedCalc Software, Ostend, Belgium.

## 3 Results

### 3.1 Patient and treatment characteristics

The median age of participants was 61,50 years (IQR: 14,50): 33 (34,38%) were women and 63 (66,62%) men. There were no significant differences in median age [62,00 years (IQR:16,00) vs. 63,00 years (IQR: 15,00); p=0,256] and BMI [27,58kg/m2 (IQR:6,52) vs. 26,29 kg/m2 (IQR:5,95); p=0,315] at the time of diagnosis between females and males. **Table 1** present population characteristic.

**Table 1:** Characteristic of population.

Characteristic populations	N=96 (100%)
Gender	
Male	63 (66,2%)
Female	33 (34,38%)
Karnofsky %	
<70	0 (0%)
70	0 (0%)
80	24 (25%)
90	53 (55%)
100	19 (20%)
RISK-MSKCC	
Favorable	36 (37%)
Intermediate	60 (63%)
Poor	0 (0%)
RISK-IMDC	
Favorable	32 (34%)
Intermediate	53 (55%)
Poor	11 (11%)
Tumor location	
Right kidney	47 (48,9%)
Left kidney	47 (48,9%)
Bilateral	2 (2,2%)
HP result	
Clear cell renal carcinoma	83(86%)
Clear cell renal carcinoma and another component (sarkomatoid)	8 (8%)
Nonclear cell renal carcinoma	5 (6%)
Classification T	
Unknown	3 (3%)
1	23 (24%)
2	11 (11%)
3	58 (60%)
4	1 (1%)
Classification N	
Unknown	28 (29%)
0	55 (57%)
1	13 (14%)
Classification M at the time of diagnosis	
Unknown	12 (13%)
0	50 (52%)
1	34 (35%)
Fuhrman scale	
1	6 (6,4%)
2	35 (36,4)
3	42 (43,7%)
4	13 (13,5%)
Number of organ involved	
To 1 organ	30 (31%)
To 2–3 organs	54 (56%)
To >3 organs	12 (13%)
First site of metastases (may exceed the group size):	
Lungs	61 (64%)
Liver	9 (9%)
Soft tissues	30 (31%)
Lymph nodes	37 (39%)
Bones	23 (24%)
Pancreas	12 (13%0
Brain	2 (2%)
Kidney on the other side	7 (7%)
Peritoneum	11 (11%)
Adrenal glands	17 (18%)
Thyroid	2 (2%)
Another (ovarium, heart, pleura, salivary gland)	5 (5%)
Best response in CT	
No data	3 (3%)
Stabilization	53 (55%)
Partial response	36 (38%)
Complete response	1 (1%)
Progression disease	3 (3%)
Reason for 1st line treatment ending	
Progression disease	60 (63%)
Toxicity	11 (11%)
Patient resignation	3 (3%)
No data/death	4 (4%)
Status on the day of end of observation	
Alive	44 (46%)
Dead	52 (64%)

T, tumor; N, lymph node; M, metastases; MSKCC scale, the Memorial Sloan Kettering Cancer Centre scale; IMDC scale, International Metastatic Renal Cell Carcinoma Database Consortium scale; HP result, histopathological result; CT, computed tomography.

### 3.2 Characteristics of treatment received

The median number of cycles of TKI administration was 11,00 (IQR: 19,00) and median therapy duration was 63 (IQR:95,00) weeks. There were no significant differences in median number of cycles and therapy duration between female and males [respectively: 15,00 (IQR: 23) vs. 10 (IQR:14); p=0,133] and 88 weeks (IQR: 133,00) vs. 60 weeks (IQR: 84,00); p=0,0123]. A positive significant correlation between OS and number of administered cycles [R=0,805; p<0,001] as well as treatment time [R=0,814; p<0,001] was confirmed. **Table 2** presents characteristic of treatment received.

**Table 2:** Characteristic of treatment received.

Characteristic treatment	Numbers 96 (percentage 100%)
Surgery treatment	
Nephrectomy	88 (92%)
Tumorectomy	7 (7%)
No surgery	1 (1%)
2 line treatment on the day of end of observation	
Yes	44 (46%)
Observation	1 (1%)
No treatment	33 (34%)
1 line of treatment continues	18 (19%)
3 line treatment on the day of end of observation	
Yes	7 (7%)
Observation	2 (2%)
No treatment	56 (58%)
1 line of treatment continues	18 (19%)
2 line of treatment continues	13 (14%)
4 line and next on the day of end of observation	
Yes	0 (0%)
Observation	2 (2%)
No treatment	59 (61%)
1 line of treatment continues	18 (19%)
2 line of treatment continues	13 (14%)
3 line of treatment continues	7 (7%)
Number of series of systemic treatment - median (range)/IQR	11 (1 -91)/19,00
Average of series of systemic treatment	18,14
Number of weeks of treatment - median (range)/IQR	63 (4 – 546)/95,00
Average number of weeks of systemic treatment	99,41

IQR, iterquartile range.

### 3.3 Types of toxicities

The most common AE were related to the gastrointestinal tract – complaints from this system were reported by 76.04% of patients. 61.46% of patients reported fatigue during the course of therapy, although this symptom was rarely reported as an isolated AE. AEs related to the cardiovascular system affected 48.96% of patients. In 81.25% of patients, AEs affecting at least two organs were observed.

64 patients (66.67%) required a dose reduction. Furthermore, 33 patients (34.38%) required a second dose reduction to sustain therapy.

#### 3.3.1 Cardiovascular toxicity

As many as 47 patients (48.96%) experienced cardiovascular toxicity during sunitynib treatment, and in 17 cases (17.71%), these toxicities were reported as dominant. The vast majority—28 patients—presented with grade 2 cardiotoxicity, followed by 10 with grade 1, 8 with grade 3, and only 1 patient developing grade 4 cardiotoxicity.

There were no significant differences in the median age or median BMI between patients with cardiotoxicities compared to those without toxicities. The respective values were 61.00 years (IQR: 15.00) versus 62.00 years (IQR: 15.00) (p=0.725), and 27.58 kg/m² (IQR: 6.52) versus 26.29 kg/m² (IQR: 5.95) (p=0.873). However, significantly more females experienced cardiotoxic effects compared to males [yes: 24/96 vs. no: 6/96; p<0.001].

A total of 17 patients (17.71%) required dose delays due to not acceptable grade 2 and severe (grade ≥3) cardiac complications, and multiple delays were necessary in 8 of these cases. Due to escalating cardiotoxic effects, 16 patients (16.67%) required dose reductions, and 6 patients (6.25%) were disqualified from further therapy.

#### 3.3.2 Hematological toxicity

Thirty-two patients (33.33%) developed hematological toxicities, including 19 cases (19.79%) where these toxicities were reported as dominant. Grade 1 hematotoxicity was reported in 2 patients, 11 patients developed grade 2 toxicity, while grade 3 toxicity was the most common, occurring in 16 cases. Only 3 patients were diagnosed with grade 4 hematotoxicity.

There were no significant differences in median age [60.00 years (IQR: 15.50) vs. 62.00 years (IQR: 12.50); p=0.635] or sex distribution [female-to-male ratio: 12/20 vs. 22/42; p=0.712] between cohorts with and without hematotoxicity. However, the median BMI of patients with hematological toxicities was significantly lower compared to those without complications [26.01 kg/m² (IQR: 5.49) vs. 27.36 kg/m² (IQR: 6.92); p=0.042].

Dose delays due to hematotoxicity were reported in 31 patients, with multiple delays required in 10 cases. Furthermore, 19 patients (19.80%) required dose reductions, and treatment was terminated in 5 patients (5.20%) due to severe hematotoxicity.

#### 3.3.3 Enterotoxicity

Toxicities involving the gastrointestinal tract were diagnosed in 73 patients (76.04%). In 49 cases (20.04%), these toxicities were reported as dominant. The vast majority of patients, 44 (45.85%), developed grade 2 enterotoxicity, followed by 12 (12.50%) with grade 3, 9 (9.37%) with grade 1, and only 2 patients (2.08%) presenting with grade 4 enterotoxicity. Patients who developed enterotoxicity were significantly younger [median age: 60.00 years (IQR: 15.00) vs. 63.00 years (IQR: 10.00); p=0.049] and had a slightly higher but not statistically significant median BMI [27.22 kg/m² (IQR: 6.41) vs. 26.00 kg/m² (IQR: 5.60); p=0.418] compared to those without enterotoxicity. There were no significant differences in gender prevalence between the analyzed cohorts [female-to-male: 27/46 vs. 6/17; p=0.435]. Dose delays due to enterotoxicity were required in 37 patients (38.54%). Dose reductions were performed in 38 patients (39.58%), and treatment was terminated in 3 patients (3.13%) due to severe side effects.

#### 3.3.4 Endocrinological toxicity

Endocrine toxicity occurred in 31 patients (32.29%), but it was reported as dominant in only 6 cases (6.25%). The vast majority, 26 patients (27.08%), developed grade 2 toxicity, while 4 (4.17%) experienced grade 1 toxicity, and only one patient (1.04%) was diagnosed with grade 3 toxicity. There were no significant differences in median age or median BMI between patients who developed endocrine toxicities compared to those who did not [61.00 years (IQR: 17.00) vs. 62.00 years (IQR: 14.00); p=0.454, and 26.33 kg/m² (IQR: 4.44) vs. 27.18 kg/m² (IQR: 6.34); p=0.604]. However, there were significantly more women than men in the cohort with endocrine toxicity [female-to-male ratio: 17/14 vs. 16/49; p=0.004]. Due to the low incidence of endocrine toxicity in patients treated with TKIs for renal cancer, dose delays were required in only 6 cases (6.25%), predominantly during the 4th series. Similarly, dose reductions were performed in only 7 cases (7.29%), and no patients required discontinuation of treatment due to endocrine toxicity.

#### 3.3.5 Dermatological toxicity

Forty-three patients (44.79%) experienced dermatological toxicity, and in 25 cases (20.04%), it was reported as dominant. A total of 35 patients (36.46%) developed grade 2 toxicity, 6 (6.25%) developed grade 1 toxicity, and only 2 (2.08%) experienced grade 3 toxicity. Dose delays were required in 24 patients (25.00%) due to toxic symptoms, which mostly occurred after the 2nd treatment series. There were no significant differences in median age or median BMI between patients who developed dermatological toxicities and those who did not [60.50 years (IQR: 15.00) vs. 62.00 years (IQR: 14.00); p=0.169, and 25.57 kg/m² (IQR: 4.92) vs. 27.50 kg/m² (IQR: 6.53); p=0.374]. Similarly, no significant differences were observed in gender distribution between the analyzed cohorts [female-to-male ratio: 27/46 vs. 6/17; p=0.887].

#### 3.3.6 Urological toxicity

Renal toxicity during sunitinib treatment was diagnosed in 24 (25,00%) patients and in 12 (12,50%) was described as dominant. Eight (8,33%) patients developed grade 1 toxicity, the next 8 (8,33%) grade 2 followed by 7 (7,29%) persons with toxicity grade 3 and the only one (1,04%) presented the 4^th^ grade renal toxicity.

Median age [63,00 years (IQR:18,00) *vs.* 61,00 years (IQR:13,00); p=0], and median BMI [26,86 kg/m^2^ (IQR:5,17) *vs.* 29,99 kg/m^2^ (IQR:6,41); p=0], did not differ significantly between patients who experienced and who did not present renal toxicities. No differences were in gender prevalence between analyzed cohorts [female-to-male: 10/14 *vs.* 23/49; p=0,385].

Dose-delay was necessarily in 15 (15,63%) patients mostly after 12^th^ series, furthermore 9 (9,36%) persons during TKI treatment required dose reduction and 2 (2,08%) had therapy termination because of severe toxicity.

#### 3.3.7 Fatigue

Fifty-nine (61,5%) participants experienced fatigue during therapy. The vast majority of i.e. 48 (50%) of patients reported fatigue during the first 3 series of TKI treatment. There were no significant differences in median age and median BMI between patients who developed and did not had fatigue [61,00 years (IQR:17,00) vs. 62,00 years (IQR:11,00); p=0,997 and 26,98 kg/m2 (IQR:4,71) vs. 26,79 kg/m2 (IQR:7,84); p=0,980]. No differences were observed in gender prevalence between analyzed cohorts [female-to-male: 22/37 vs. 11/26; p=0,448].

Detailed analysis of toxicities is showed in **Tables 3A–G**.

**Table 3A:** Detailed analysis of cardiotoxicity.

Cardiotoxicity	Overall 96 (100%)
No	49 (51%)
Yes	47 (48.96%)
Hypertension	37 (38.54%)
ischemic heart disease/heart attack	0 (0%)
Heart failure	6 (6.25%)
Thromboembolic events	9 (9.37%)
Arrhythmias	12 (12.5%)
Cardio-vascular toxicity grade	
Grade 1	10 (10.41%)
Grade 2	28 (29.16%)
Grade 3	8 (8.33%)
Grade 4	1 (1.04%)
Time to start cardio-vascular system toxicity	
No	49 (51%)
Between series 1 and 3	33 (34.76%)
Between series 3 and 8	13 (13.54%)
Between series 8 and 12	2 (2.08%)
After series 12	9 (9.37%)
Because of cardio-vascular system toxicity delay in treatment	
No	75 (78.12%)
Yes	17 (21.86%)
Because of cardio-vascular system toxicity dose reduction	
No	74 (77.08%)
Yes	16 (16.67%)
Because of cardio-vascular system toxicity end of treatment	6 (6.25%)

**Table 3B:** Detailed analysis of hematological toxicity.

Hematological toxicity:	Overall 96 (100%)
No	64 (66.69%)
Yes	32 (33.33%)
Anemia	11(11.45%)
neutropenia	18 (18.75%)
Thrombocytopenia	8 (8.33%)
Hematological toxicity grade	
Grade 1	2 (2.08%)
Grade 2	11(11.45%)
Grade 3	16 (16.67%)
Grade 4	3 (3.12%)
Time to start hematological toxicity	
No	64 (66.69%)
Between series 1 and 3	16 (16.67%)
Between series 3 and 8	19 (19.80%)
Between series 8 and 12	6 (6.25%)
After series 12	4 (4,16%)
Because of hematological toxicity delay in treatment	
No	65 (67.70%)
Yes	31 (32.29%)
Because of hematological toxicity dose reduction	
No	72 (75%)
Yes	21 (21.88%)
Because of hematological toxicity end of treatment	5 (5.20%)

**Table 3C:** Detailed analysis of gastrointestinal toxicity.

Enterotoxicity	Overall 96 (100%)
No	23 (23,96%)
Yes	73 (76.04%)
Nausea/heartburn	15 (15.62%)
Vomiting	6 (6.25%)
Lost of appetite/dysgeusia	29 (30.20%)
Diarrhea	41 (42.70%)
Stomatitis	29 (30.20%)
Hepatotoxicity	21 (21.88%)
Other	9 (9.37%)
Digestive system toxicity grade	
Grade 1	9 (9.37%)
Grade 2	44 (45.85%)
Grade 3	12 (12.5%)
Grade 4	2 (2.08%)
Time to start digestive system toxicity	
No	23 (23.96%)
Between series 1 and 3	49 (51.03%)
Between series 3 and 8	21 (21.88%)
Between series 8 and 12	9 (9.37%)
After series 12	9 (9.37%)
Because of digestive system toxicity delay in treatment	
No	59 (61.46%)
Yes	37 (38.54%)
Because of digestive system toxicity dose reduction	
No	55 (57.29%)
Yes	38 (39.58%)
Because of digestive system toxicity end of treatment	3 (3.12%)

**Table 3D:** Detailed analysis of endocrinological toxicity.

Endocrinological toxicity	Overall 96 (100%)
No	65 (67.71%)
Yes	31 (32.29%)
Hypothyroidism	30 (31.25%)
Hyperthyroidism	2 (2.08%)
Adrenal gland disorders	0 (0%)
Hyperglycemia	1 (1.04%)
Other	0 (0%)
Endocrinological toxicity grade	
Grade 1	4 (4.2%)
Grade 2	26 (27.09%)
Grade 3	1 (1.04%)
Grade 4	0 (0%)
Time to start endocrinal toxicity	
No	65 (67.71%)
Between series 1 and 3	9 (9.37%)
Between series 3 and 8	22 (22.91%)
Between series 8 and 12	2 (2.08%)
After series 12	4 (4.16%)
Because of endocrinal system toxicity delay in treatment	
No	90 (93.75%)
Yes	6 (6.25%)
Because of endocrinal system toxicity dose reduction	
No	89 (92.71%)
Yes	7 (7.29%)
Because of endocrinal toxicity end of treatment	0 (0%)

**Table 3E:** Detailed analysis of dermatological toxicity.

Dermatological toxicity	Overall 96 (100%)
No	53 (55.21%)
Yes:	43 (44.79%)
Hand-foot syndrome	27 (28.12%)
Rash	14 (14.58%)
Conjunctivitis	6 (6.25%)
Changes in hair color	11(11.45%)
Yellow/red skin	8 (8.33%)
Other	9 (9.37%)
Skin toxicity grade	
Grade 1	6 (6.25%)
Grade 2	35 (36.46%)
Grade 3	2 (2.08%)
Grade 4	0 (0%)
Time to start skin toxicity	
No	53 (55.21%)
Between series 1 and 3	24 (25%)
Between series 3 and 8	15 (15.62%)
Between series 8 and 12	6 (6.25%)
after series 12	4 (4.16%)
Because of skin toxicity delay in treatment	
No	72 (75%)
Yes	24 (25%)
Because of skin toxicity dose reduction	
No	75 (78.12%)
Yes	20 (20.84%)
Because of skin toxicity end of treatment	1 (1.04%)

**Table 3F:** Detailed analysis of renal toxicity.

Urological toxicity	Overall 96 (100%)
No	72 (75%)
Yes	24 (25%)
Kidney failure	18 (18.75%)
Proteinuria	10 (10.41%)
Urinary tract infection	4 (4.16%)
Other	0 (0%)
Renal toxicity grade	
Grade 1	8 (8.33%)
Grade 2	8 (8.33%)
Grade 3	7 (7.29%)
Grade 4	1 (1.04%)
Time to start renal toxicity	
No	72 (75%)
Between series 1 and 3	7 (7.29%)
Between series 3 and 8	7 (7.29%)
Between series 8 and 12	4 (4.16%)
After series 12	11 (11.45%)
Because of renal toxicity delay in treatment	
No	81 (84.36%)
Yes	15 (15.64%)
Because of renal toxicity dose reduction	
No	85 (88.54%)
Yes	9 (9.37%)
Because of renal toxicity end of treatment	2 (2.08%)

**Table 3G:** Detailed analysis of fatigue.

Fatigue	Overall 96 (100%)
No	37 (38.54%)
Yes	59 (61.46%)
Time to start fatigue	
No	37 (38.54%)
Between series 1 and 3	48 (50%)
Between series 3 and 8	5 (5.2%)
Between series 8 and 12	4 (4.2%)
After series 12	2 (2.08%)
Because of fatigue dose reduction	
No	70 (73%)
Yes	23 (24%)
Because of fatigue stop treatment	3 (3.12%)

### 3.4 Impact of toxicities on survival

**Figure 1:**
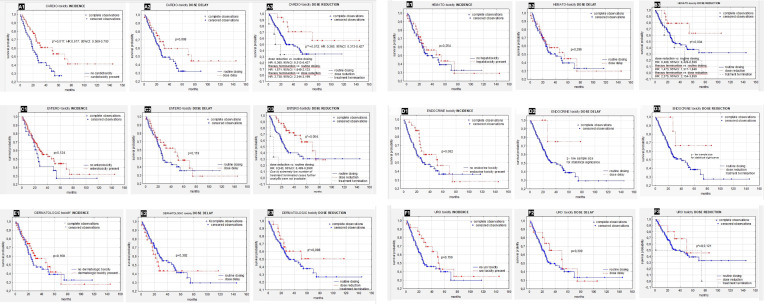
**(A-F)** Survival in patients with ccRC depending on types of toxicity: **(A1)** Impact of cardiotoxicity on survival (p=0,017 and HR:0,617; 95%CI: 0,569-0,700). **(A2)** Impact of dose- delay due to cardiotoxicity on survival. **(A3)** Impact of dose reduction due to cardiotoxicity on survival (p=0,012 and HR: 0,365; 95%CI: 0,313-0,42). **(B1)** Impact of hematologic toxicities on survival. **(B2)** Impact of dose- delay due to hematologic toxicities on survival. **(B3)** Impact of dose reduction due to hematologic toxicities on survival. **(C1)** Impact of entero-toxicity on survival. **(C2)** Impact of dose- delay due to entero-toxicity on survival. **(C3)** Impact of dose reduction due to entero-toxicity on survival (p=0,004 and HR: 0,543; 95%CI: 0,499-0,590). **(D1)** Impact of endocrine toxicity on survival. **(D2)** Impact of dose- delay due to endocrine toxicity on survival. **(D3)** Impact of dose reduction due to endocrine toxicity on survival. **(E1)** Impact of dermatologic toxicity on survival. **(E2)** Impact of dose- delay due to dermatologic toxicity on survival. **(E3)** Impact of dose reduction due to dermatologic toxicity on survival. **(F1)** Impact of urological toxicity on survival. **(F2)** Impact of dose- delay due to urological toxicity on survival. **(F3)** Impact of dose reduction due to urological toxicity on survival. ccRC, clear cell renal cancer; HR, Hazard Ratio; CI, 95% confidence interval. *p<0.05 is statistically significant.

Patients who developed cardiotoxicity showed significantly better survival compared to person with no cardiotoxicity (p=0,017 and HR:0,617; 95%CI: 0,569-0,700)) (**Figure 1A1**). There was no defenses in survival between patients who had dose-delay compared to ones with routine dosing (p=0,068) (**Figure 1A2**). Six (6,25%) patients required therapy termination due to severe cardiotoxicity and were excluded from further survival analysis due to small sample size. Additionally analysis showed that reduction of sunitynib dose (but not treatment termination) due to cardiotoxic complications improved survival significantly (**Figure 1A3**) (p=0,012 and HR: 0,365; 95%CI: 0,313-0,427). Furthermore ones who required therapy termination due to severe cardiac side effects showed significantly worse survival compared to patients without dose reduction (HR: 1,871; 95%CI: 1,649-2,123) as well compared to participants with dose reduction (HR: 2,738; 95%CI: 2,344-3,199) (**Figure 1A3**).

In contrast to cardiotoxicity, persons who experienced the presence of hematologic toxicities did not showed better survival compared to the ones who did not (**Figure 1B1**) (p=0,254). Similarly dose delay due to hematologic toxicity that occurred during the therapy did not affect survival (**Figure 1B2**) (p=0,296). However, patients who underwent dose reduction due to hematologic adverse events performed significantly better survival compared to participants without hematologic toxicity (**Figure 1B3**) (p=0,004 and HR: 0,488; 95%CI: 0,426-0,559). Counterrally patients who required therapy termination due to hematologic adverse effects showed the worse survivals compared to ones without dose reduction (HR: 1,473; 95%CI: 1,317-1,648) (**Figure 1B3**).

No significant differences were reported in OS if compared patients with and without entero- toxicity (**Figure 1C1**) (p=0,124) nightery between persons who had dose delay due compared to those who did not (**Figure 1C2**) (p=0,119). Patients who required dose reduction due to gastrointestinal toxicity symptoms showed significantly better survival during therapy compared to the ones who did not presented gastrointestinal toxicity (**Figure 1C3**) (p=0,004 and HR: 0,543; 95%CI: 0,499-0,590). Tree (3,13%) patients had terminated therapy due to sever hepatotoxic complications and were not included in the further dose reduction analysis due to extremely small sample size.

Survival did not differ significantly between patients who experienced endocrine toxicity during TKI therapy compared to the persons who did not (**Figure 1D1**) (p=0,082). Although survival curves for patients with dose delay or dose reduction show some visual divergence, the analysis did not demonstrate statistically significant differences. Given the small sample size and lack of p-values, no conclusions can be drawn regarding survival impact (**Figures 1D2** **,** **1D3**).

Persons who experienced dermatological toxicities during sunitinib therapy did not show better overall survival (**Figure 1E1**) (p=0,160). Similarly, delayed dosing due to toxic symptoms did not impact the patients’ overall survival (**Figure 1E2**). Patients who required dose reduction during TKI therapy showed better survival, however the difference was insignificant (**Figure 1E3**) (p=0,098).

Survival of renal cancer patients treated with TKI was influenced neither by presence of urological toxicity nor by delayed dosing due to it (**Figures 1F1**, **1F2**) (p=0,150 and p=0,309). Similarly comparable survival of patients who experienced dose reduction during therapy compared to persons with routine dosing were observed (**Figure 1F3**) (p=0,121).

**Figure 2:**
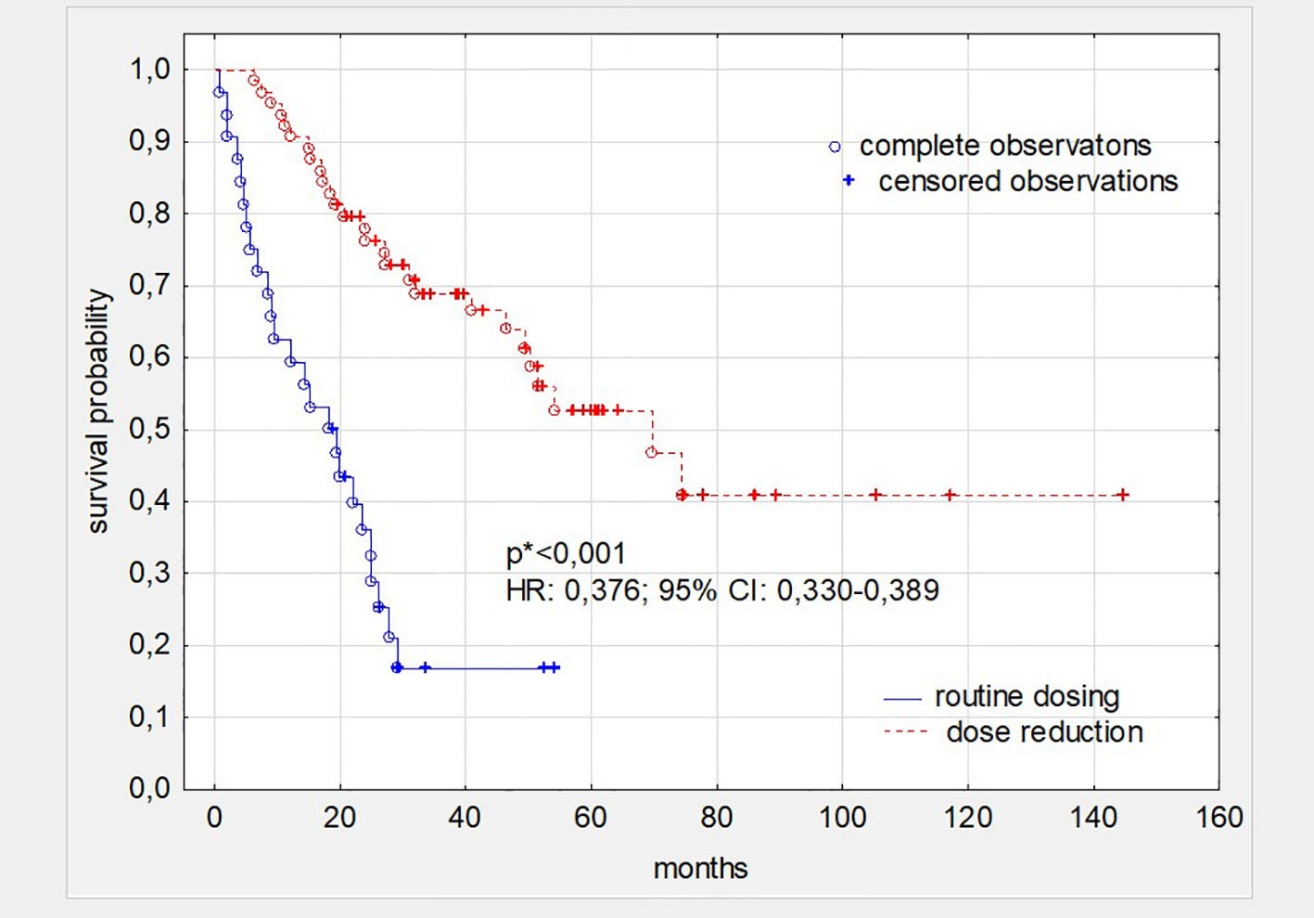
Survival in patients with ccRC depending on dosing. *the level of statistical significance was calculated comparing patients’ cohorts with single and double dose reduction (p<0,001 and HR: 0,376; 95% CI: 0,330-0,389). ccRC, clear cell renal cancer; HR, Hazard Ratio; CI, 95% confidence interval. *p<0.05 is statistically significant.

**Figure 3:**
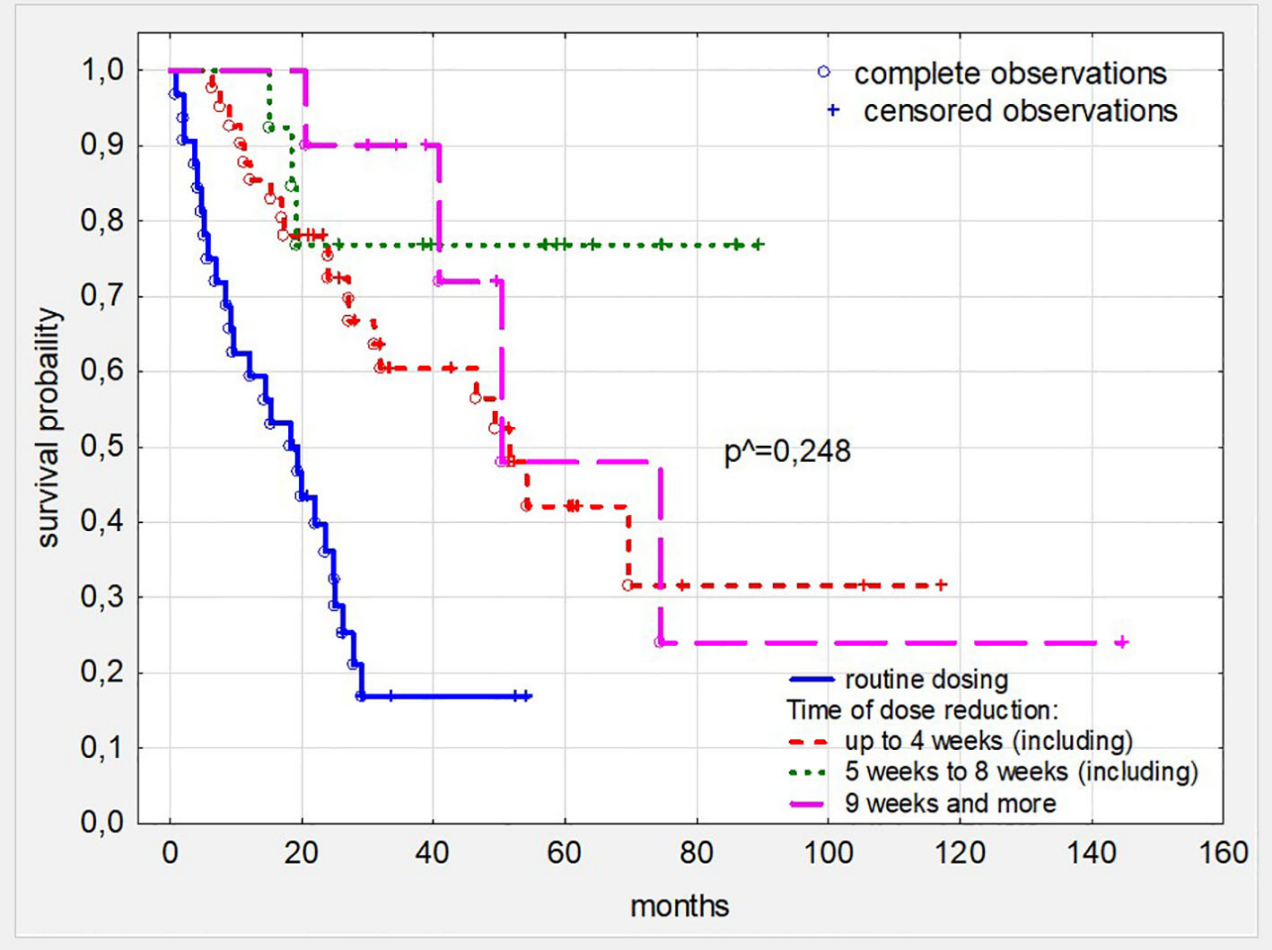
Survival in patients with ccRC depending on the time the dose reduction was introduced. ^ - the level of statistical significance was calculated comparing patients’ cohorts with dose reduction only. ccRC, clear cell renal cancer.

**Figure 4:**
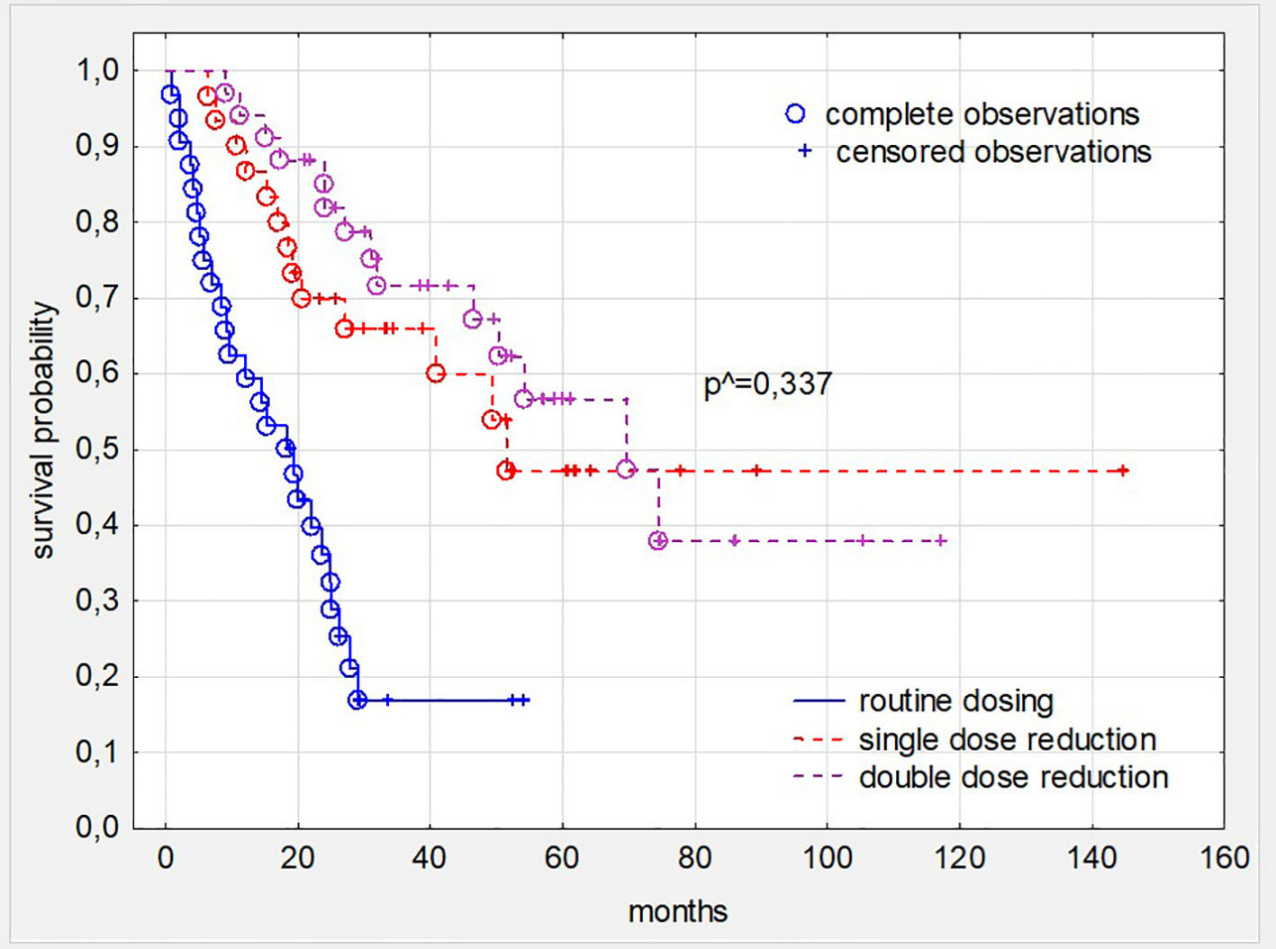
Survival in patients with ccRC depending on dosing schedule and number of required dose reduction due to all toxic symptoms. ^ - the level of statistical significance was calculated comparing patients’ cohorts with single and double dose reduction. ccRC, clear cell renal cancer.

Subsequent, comprehensive analysis of the impact of all toxicities resulting from sunitynib therapy showed that patients who required at least one dose reduction, but not treatment termination during TKI treatment had better survival (p<0,001 and HR: 0,376; 95%CI: 0,330-0,389) (**Figure 2**). The time in which the dose reduction was defined as early (reduction after 1–3 series), medium (reduction after 4–8 series) and long (reduction after 9 series or more) did not significantly affect the survival time of patients treated with this TKI (**Figure 3**). What is also an interesting finding survival of patients who had only one dose reduction If compared to survival of persons who had reduced dose twice also did not reveal significant difference (**Figure 4**).

## 4 Discussion

The study highlights the incidence of organ toxicities associated with sunitynib in advanced RCC, focusing on their impact on OS and treatment duration.

Consistent with prior reports, toxicities emerge as potential biomarkers of sunitinib efficacy. The findings also emphasize the need for individualized treatment and careful toxicity monitoring to optimize outcome.

Cardiotoxicity arises from off-target effects on cardiovascular pathways. VEGFR inhibition by TKIs disrupts endothelial function, reduces nitric oxide, and increases vascular resistance, leading to hypertension and other complications. Direct myocardial toxicity and mitochondrial dysfunction are also implicated (6, 13). In our study, cardiovascular toxicities occurred in 49.0% of sunitinib -treated patients, with hypertension (38.54%) being most common, followed by arrhythmias (12.5%), thromboembolic events (9.37%), and heart failure (6.25%). Severe toxicities (Grade ≥3) affected 9.37%%, leading to dose delays in 21.86%, reductions in 16.67%, and discontinuation in 6.25%. Our 38% hypertension rate aligns with the reported 15%-49% range in the literature (10). Arrhythmias and heart failure, though less common, are significant, with rates of 12% and 7% reported by Rautiola et al., similar to ours (27). Thromboembolic events (9.37% of our patients) align with the 5%-10% reported in the literature (6). Incidence variations may reflect patient demographics, cardiovascular risk, and monitoring. Most toxicities in our study were Grade 2 (20.84%) or 3 (12.5%), consistent with data showing manageable hypertension but severe cases requiring dose adjustments or discontinuation (27, 28). Early cardiotoxicity management is vital for treatment adherence and outcomes (6). In our study, cardiotoxicity was linked to improved survival (p=0.017), with dose reductions further enhancing OS (p=0.012), suggesting cardiotoxicity as a marker of VEGFR inhibition. Similar studies associate hypertension with prolonged PFS and OS (27). Donskov et al. found sunitinib-induced hypertension linked to longer PFS (14 vs. 7 months, p=0.001) and OS (30 vs. 16 months, p=0.002) (13). Our study and published data support cardiotoxicity as a biomarker of efficacy, highlighting the need for proactive management and individualized treatment based on patient risk and toxicity profiles.

As a result of the action of TKIs, there is a blockade of many tyrosine kinase receptors, including the blockade of VEGFR, FMS-like tyrosine kinase 3 (FLT-3) and c-kit receptors, which promote hematopoiesis (29, 30). Additionally, in the case of the c-kit receptor, blockade occurs in hematopoietic stem cells (31). A side effect of chronic TKI use is myelosuppression (32), which manifests as anemia, leukopenia, neutropenia, and thrombocytopenia (32). In our analysis, hematological toxicity occurred in 33.33% of patients. The most common complication was neutropenia, affecting 18.75% of patients. Anemia occurred in 11.45% of patients and, thrombocytopenia affected 9.37% of patients. Most toxicities were Grade 2 or 3 (28.12%). Kumar and al. in their analysis showed, that the most often hematological toxicity was neutropenia - observed in 72% of patients treated with sunitinib and the next was thrombocytopenia, which was observed in 65% of patients (29). Hong et al. found in their analysis that the use of sunitinib is associated with the occurrence of anemia in 69.7% of patients, thrombocytopenia in 77.6%, and neutropenia in 71.1% (33) Donskov et al. noted in their analysis, that neutropenia is significantly associated with both PFS and OS, thrombocytopenia were not significantly associated with either PFS or OS (13).

However in our study we did not noticed, that persons who experienced the presence of hematologic toxicities showed better survival compared to the ones who did not.

We observed, enterotoxicity in 76.04% of patients, with diarrhea, stomatitis, and appetite loss/dysgeusia being the most frequent symptoms. These findings align with Porta et al., who identified diarrhea, stomatitis and mucosal inflammation as common AE in sunitinib-treated patients (34), and Arena et al., who reported stomatitis in 35% of sunitinib-treated patients, mostly of Grade 1–2 severity (35). Most toxicities in our study were mild to moderate, with severe (Grade ≥3) events being rare (15%). Treatment delays occurred in 38.54% of patients and dose reductions in 39.58%. However, no significant differences in OS were found between patients with and without gastrointestinal toxicity or those experiencing dose delays, consistent with Porta et al., who noted no cumulative effect of gastrointestinal toxicities on long-term outcomes (34). Hepatotoxicity was observed in 21.88% of our patients, with Grade 3–4 toxicity in 5 patients (5.2%). In our study hepatotoxicity led to dose reductions in 11.45% of patients and treatment discontinuation in 3.12%.

Hypothyroidism, a common TKI toxicity in advanced RCC, arises from multifactorial mechanisms, including cytotoxicity to thyroid cells, thyroid peroxidase inhibition, disrupted iodine uptake, altered hormone metabolism, and reduced vascularization via VEGFR inhibition (36) The prevalence of Grade 1 and Grade 2 endocrinological toxicities in our study aligns with the lower range of reported rates, with Wu and Huang noticed a 24%-85% incidence across studies with varying methodologies (36). Vasileiadis et al. and Badran et al. reported hypothyroidism rates of 40% and 40.3%, consistent with our findings (37, 38). Bozkurt et al. and Tassi et al. observed slightly higher rates of 42.3% and 45.8%, respectively [39.40]. The differences, though not significant, likely reflect variations in patient characteristics, treatments, and monitoring. Grade 2 hypothyroidism was most common (27.09%), with one Grade 3 case and none of Grade 4, consistent with the typically mild-to-moderate nature reported in the literature (36, 40). Hypothyroidism is manageable with hormone replacement, enabling treatment continuation (36). In our study, endocrine toxicities, including hypothyroidism, showed no significant correlation with OS or PFS. In contrast, Bozkurt et al. reported significantly longer PFS (14 vs. 6 months) and OS (30 vs. 12 months, p=0.001) in patients with sunitinib-induced hypothyroidism (39). Vasileiadis et al. reported a median OS of 32 months in hypothyroid patients vs. 15 months in euthyroid individuals (p=0.03) (37). Buda-Nowak et al. and Badran et al. linked hypothyroidism to improved TKI efficacy, with longer PFS and OS (16, 38). Regular monitoring and management of sunitinib induced hypothyroidism are vital for optimizing outcomes through individualized care.

In our study, 44.79% of patients experienced dermatological toxicity, with HFS (28.12%) and rash (14.58%) being the most common, alongside hair color changes (11.45%). Lee et al. reported HFS in 36% and rash in 20% of sunitinib-treated patients (41). Most toxicities were Grade 1 or 2, with Grade 2 predominating (36.46%), and severe (Grade ≥3) toxicities being rare (2.08%). Treatment delays were noted in 25%, and dose reductions in 20.84%. No significant survival impact was found. Poprach et al. observed better PFS (20.8 vs. 11.1 months, p=0.007) and OS (43.0 vs. 31.0 months, p=0.027) in sunitinib-treated patients with skin toxicities (15).

Early detection and management of these toxicities are critical for maintaining adherence and efficacy, supporting their potential as biomarkers of treatment outcomes.

As a result of using TKIs in patients with mRCC, kidney damage may occur. The causes of this phenomenon may include endothelial damage, secondary autoimmune disorders, or dehydration induced by TKIs (42, 43), but the exact mechanisms of this process are not fully understood (42). The most common adverse effects resulting from kidney damage during TKI treatment are renal function failure and proteinuria (44). However, it should be noted that approximately 50% of patients with mRCC have a reduced glomerular filtration rate (eGFR) before starting treatment (45) or may have proteinuria (46). The presence of these disorders is not an absolute contraindication to the use of TKI therapy (46). In our study 25% of patients experienced renal toxicity, with kidney failure (18.75%) and proteinuria (10.41%). Moreover, 4.16% of patients were diagnosed with urinary tract infection. Toxicities were Grade 1, Grade 2 or Grade 3 at a similar level (23). In our study 76% of patients had increased eGFR before the start of treatment.: In COMPARZ trial an increase in creatinine was observed in 46% during treatment with sunitinib (12). Similar results were presented in the analysis by Mielczarek et al. during sunitinib therapy, an increase in creatinine was recorded in the range of 7.7% to 33% of patients (47). In the analysis conducted by Gupta et al., renal insufficiency was observed in 33% of patients with mRCC treated with TKIs (48). In a meta-analysis presented by Ren et al. kidney injury occurred in approximately 17%, and proteinuria in 29% of patients treated with TKIs. Most adverse events were graded 1 or 2 according to CTCAE (44). Our analysis did not show an impact of renal toxicity on the overall survival results of the studied group of patients. Similar conclusions were reached in the analyses by Mielczarek et al. and Gupta et al., who found that patients with mRCC and renal function impairment treated with anti-VEGF drugs did not differ in terms of response rates, time to treatment failure, and overall survival from patients with normal renal function (47, 48). Macfarlane et al. also noted that reduced eGFR did not affect objective response or overall survival (45). Additionally, Kato et al. in their meta-analysis found that the occurrence or worsening of proteinuria did not show a significant association with a higher risk of death and renal failure. This suggests that anti-angiogenic drugs can be administered to patients regardless of their proteinuria status, although careful assessment of the benefits and harms of this therapy is necessary (46).

An additional element that we assessed in our study was the determination of the frequency of fatigue and the impact of this parameter on treatment outcomes. The causes of fatigue are attributed to symptoms related to cancer, the side effects of cancer therapies, comorbidities of the patient, or psychosocial factors, but the mechanism of fatigue’s onset is not fully understood (49). It is considered one of the main factors influencing treatment effectiveness (49). Fatigue rarely occurs as a singular symptom (50, 51). In our analysis, fatigue was reported by 59 patients (61.46%). Fatigue most commonly occurred during the first 3 cycles of treatment. The occurrence of fatigue alone was not a reason for dose reduction or treatment delay. However, if it occurred alongside symptoms from other organs, dose reduction was implemented in 23 patients (24%). Treatment was discontinued in 3 individuals, but this was not the only reason for discontinuation; it was a contributing factor. In meta-analysis published by Santoni et al., it was shown that fatigue is one of the most commonly reported adverse effects of TKIs (52). In the COMPARZ study, fatigue was noted in 63% of patients using sunitinib (12), while in the study by Ekenel et al., fatigue occurred in 59% of patients (53). Cancer-related fatigue may lead to dose reduction, treatment delays, or early termination of therapy, which can negatively impact treatment outcomes (51). However, Donskov et al. in their analysis showed that the occurrence of fatigue was not significantly associated with either PFS or OS (13).

In phase 3 clinical studies evaluating the efficacy of combination therapies involving immune checkpoint inhibitors (ipilimumab + nivolumab (54)) or combinations of a checkpoint inhibitor with a kinase inhibitor (pembrolizumab + lenvatinib (55), pembrolizumab + everolimus (55), nivolumab + cabozantinib (56), pembrolizumab + axitinib (57)) as first-line treatment for advanced renal cell carcinoma, sunitinib served as the comparator (54–57). In these studies, the secondary endpoint was the safety of the investigated therapy compared to the control medication (54–57). In the CheckMate 214 study, adverse events (AEs) of any grade occurred in 97% of patients treated with sunitinib, and treatment was discontinued due to AEs in 12% of them (54). In the CLEAR study, 98.5% of patients treated with sunitinib experienced any AEs, and AEs of any grade led to discontinuation of the drug in 14.4% of cases (55). In the CheckMate 9ER study, adverse events (AEs) of all severities were observed in 99.1% of patients treated with sunitinib, with 16.9% discontinuing treatment due to AEs (56). Similarly, in the KEYNOTE-426 trial, any-grade AEs were reported in 99.5% of patients receiving sunitinib, leading to discontinuation in 13.9% of cases (57). In each of these studies, treatment discontinuation due to AEs was more common in the experimental groups (54–57).

Recently, there has been an increasing number of publications concerning the optimization of TKI dosage. Several studies have confirmed that low doses of TKIs can effectively maintain clinical response (58). In our analysis, we demonstrated that reducing the dose, regardless of which stage of therapy it occurred, extended the survival time of patients whose doses were decreased. The doses were reduced in 66,67% of patients. According to Powles et al., the dose reduction applied to 26% of patients treated with sunitinib (59). And In the COMPARZ study, dose reduction was observed in 51% of patients treated with sunitinib (12). However, none of these studies analyzed the impact of dose reduction on survival. An alternative method of use sunitinib: 2 weeks on/1 week off dosing schedule, reported to improve tolerability (60–62), was not used in our cohort due to reimbursement limitations at that time, but may represent a viable option in clinical practice.

The apparent similarity in survival between patients with dose delay and those with dose reduction may be explained by the frequent overlap between these subgroups, as many patients who experienced a delay in treatment subsequently required dose reduction.

## Study limitations

This study has several limitations. Its retrospective design may introduce bias, though data from a structured clinical registry ensure reliability. The single-center setting limits generalizability, but as a high-volume cancer care institution, the findings reflect real-world conditions. The sample size (96 patients) limits subgroup analyses, yet the study provides valuable insights into treatment outcomes and toxicity management. The lack of quality-of-life data precludes a full assessment of toxicity impact, though tolerability was inferred from dose modifications and treatment delays. The three-year follow-up may omit long-term outcomes but captures key therapeutic and toxicity data. This approach was dictated by the limited sample size and the need to asses overall sunitinib toxicity trend. Additionally, in clinical practice, the choice between TKIs is often influenced by factors such as physician preference, patient tolerance, and institutional protocols rather than clear evidence favoring one over the other in terms of toxicity.

Despite these limitations, the study offers meaningful real-world insights into sunitinib use in advanced RCC.

## 5 Conclusions

This study explores the incidence and impact of sunitynib-induced organ toxicities in advanced RCC, focusing on treatment adherence, dose modifications, and survival in real-world settings. By examining toxicities like cardiotoxicity, it aims to clarify their prognostic significance and inform strategies for optimizing patient management and therefore improve patient outcomes, including reduction of mortality in working-age patients which can reduce productivity loss (17). Moreover in our analysis, we demonstrated that reducing the dose, but not treatment cessation due to AE regardless of which stage of therapy it occurred, extended the survival time of patients whose doses were decreased.

## Data Availability

The raw data supporting the conclusions of this article will be made available by the authors, without undue reservation.

## References

[1] Bray F Laversanne M Sung H Ferlay J Siegel RL Soerjomataram I. Global cancer statistics 2022: GLOBOCAN estimates of incidence and mortality worldwide for 36 cancers in 185 countries. CA Cancer J Clin. (2024) 74:229–63. doi: 10.3322/caac.21834, PMID: 38572751

[2] Hsieh JJ Purdue MP Signoretti S Swanton C Albiges L Schmidinger M. Renal cell carcinoma. Nat Rev Dis Primers. (2017) 3:17009. doi: 10.1038/nrdp.2017.9, PMID: 28276433 PMC5936048

[3] Michalak M Kopczyńska A Antczak A Milecki T Tomczak P. Outcomes of treatment, laboratory results, adverse effects, and tolerability of cancer treatment in patients with metastatic renal cell carcinoma treated with ipilimumab and nivolumab after cytoreductive nephrectomy. NOWOTWORY J Oncol. (2024) 74:344–50. doi: 10.5603/njo.102357

[4] Furge KA Lucas KA Takahashi M Sugimura J Kort EJ Kanayama HO. Robust classification of renal cell carcinoma based on gene expression data and predicted cytogenetic profiles. Cancer Res. (2004) 64:4117–21. doi: 10.1158/0008-5472.CAN-04-0534, PMID: 15205321

[5] Clark PE. The role of VHL in clear-cell renal cell carcinoma and its relation to targeted therapy. Kidney Int. (2009) 76:939–45. doi: 10.1038/KI.2009.296, PMID: 19657325 PMC2963106

[6] Grela-Wojewoda A Pacholczak-Madej R Adamczyk A Korman M Püsküllüoğlu M. Cardiotoxicity induced by protein kinase inhibitors in cancer patient population. Int J Mol Sci. (2022) 23(5):2815. doi: 10.3390/ijms23052815, PMID: 35269958 PMC8910876

[7] Powles T Albiges L Bex A Comperat E Grünwald V Kanesvaran R. Renal cell carcinoma: ESMO Clinical Practice Guideline for diagnosis, treatment and follow-up. Ann Oncol. (2024) 35:692–706. doi: 10.1016/j.annonc.2024.05.537, PMID: 38788900

[8] Amaro F Pisoeiro C Valente MJ Bastos M de L Guedes de Pinho P Carvalho M. Sunitinib versus Pazopanib Dilemma in Renal Cell Carcinoma: New Insights into the *In Vitro* Metabolic Impact, Efficacy, and Safety. Int J Mol Sci. (2022) 23(17):9898. doi: 10.3390/IJMS23179898/S1, PMID: 36077297 PMC9456255

[9] Motzer RJ Hutson TE McCann L Deen K Choueiri TK. Overall survival in renal-cell carcinoma with pazopanib versus sunitinib. N Engl J Med. (2014) 370:1769–70. doi: 10.1056/NEJMC1400731, PMID: 24785224

[10] Sternberg CN Davis ID Mardiak J Szczylik C Lee E Wagstaff J. Pazopanib in locally advanced or metastatic renal cell carcinoma: results of a randomized phase III trial. J Clin Oncol. (2010) 28:1061–8. doi: 10.1200/JCO.2009.23.9764, PMID: 20100962

[11] Escudier B Porta C Bono P Powles T Eisen T Sternberg CN. Randomized, controlled, double-blind, cross-over trial assessing treatment preference for pazopanib versus sunitinib in patients with metastatic renal cell carcinoma: PISCES Study. J Clin Oncol. (2014) 32:1412–8. doi: 10.1200/JCO.2013.50.8267, PMID: 24687826

[12] Motzer RJ Hutson TE Cella D Reeves J Hawkins R Guo J. Pazopanib versus sunitinib in metastatic renal-cell carcinoma. New Engl J Med. (2013) 369:722–31. doi: 10.1056/NEJMOA1303989/SUPPL_FILE/NEJMOA1303989_DISCLOSURES.PDF 23964934

[13] Donskov F Michaelson MD Puzanov I Davis MP Bjarnason GA Motzer RJ. Sunitinib-associated hypertension and neutropenia as efficacy biomarkers in metastatic renal cell carcinoma patients. Br J Cancer. (2015) 113:1571. doi: 10.1038/BJC.2015.368, PMID: 26492223 PMC4705883

[14] Rini BI Cohen DP Lu DR Chen I Hariharan S Gore ME. Hypertension as a biomarker of efficacy in patients with metastatic renal cell carcinoma treated with sunitinib. JNCI J Natl Cancer Inst. (2011) 103:763. doi: 10.1093/JNCI/DJR128, PMID: 21527770 PMC3086879

[15] Poprach A Pavlik T Melichar B Puzanov I Dusek L Bortlicek Z. Skin toxicity and efficacy of sunitinib and sorafenib in metastatic renal cell carcinoma: a national registry-based study. Ann Oncol. (2012) 23:3137–43. doi: 10.1093/ANNONC/MDS145, PMID: 22700990

[16] Buda-Nowak A Kucharz J Dumnicka P Kuzniewski M Herman RM Zygulska AL. Sunitinib-induced hypothyroidism predicts progression-free survival in metastatic renal cell carcinoma patients. Med Oncol. (2017) 34(4):68. doi: 10.1007/S12032-017-0928-Z, PMID: 28343336 PMC5366170

[17] Baldazzi V Tassi R Lapini A Santomaggio C Carini M Mazzanti R. The impact of sunitinib-induced hypothyroidism on progression-free survival of metastatic renal cancer patients: a prospective single-center study. Urol Oncol. (2012) 30:704–10. doi: 10.1016/J.UROLONC.2010.07.015, PMID: 20884255

[18] Seweryn M Banas T Augustynska J Lorenc O Kopel J Pluta E. The direct and indirect costs of breast cancer in Poland: estimates for 2017–2019. Int J Environ Res Public Health. (2022) 19:16384. doi: 10.3390/ijerph192416384, PMID: 36554267 PMC9778099

[19] Brierley JD GMWC. TNM classification of Malignant tumors(UICC-TNM. In: Wittekind C, editor. Union for International Cancer Control (Union Internationale Contre le Cancer; UICC) under the name of TNM Classification of Malignant Tumors(UICC-TNM), 8thedn ed. John Wiley and Sons, Chichester (2017). p. 199–202.

[20] Motzer RJ Bacik J Mariani T Russo P Mazumdar M Reuter V. Treatment outcome and survival associated with metastatic renal cell carcinoma of non-clear-cell histology. J Clin Oncol. (2002) 20:2376–81. doi: 10.1200/JCO.2002.11.123, PMID: 11981011

[21] Schwartz LH Litière S De Vries E Ford R Gwyther S Mandrekar S. RECIST 1.1-Update and clarification: From the RECIST committee. Eur J Cancer. (2016) 62:132–7. doi: 10.1016/J.EJCA.2016.03.081, PMID: 27189322 PMC5737828

[22] Heng DYC Xie W Regan MM Harshman LC Bjarnason GA Vaishampayan UN. External validation and comparison with other models of the International Metastatic Renal-Cell Carcinoma Database Consortium prognostic model: a population-based study. Lancet Oncol. (2013) 14:141–8. doi: 10.1016/S1470-2045(12)70559-4, PMID: 23312463 PMC4144042

[23] Available online at: https://ctep.cancer.gov/protocoldevelopment/electronic_applications/docs/ctcae_v5_quick_reference_5x7.pdf (Accessed June 29, 2025).

[24] Available online at: https://ec.europa.eu/health/documents/community-register/2016/20161109136193/anx_136193_pl.pdf (Accessed June 29, 2025).

[25] Version 3.2025 ^©^ 2025 National Comprehensive Cancer Network^©^ (NCCN^©^). Available online at: https://www.nccn.org/professionals/physician_gls/pdf/kidney_blocks.pdf (Accessed June 29, 2025).

[26] Motzer RJ Hutson TE Tomczak P Michaelson MD Bukowski RM Rixe O. Sunitinib versus interferon alfa in metastatic renal-cell carcinoma. N Engl J Med. (2007) 356:115–24. doi: 10.1056/NEJMOA065044, PMID: 17215529

[27] Rautiola J Donskov F Peltola K Joensuu H Bono P. Sunitinib-induced hypertension, neutropenia and thrombocytopenia as predictors of good prognosis in patients with metastatic renal cell carcinoma. BJU Int. (2016) 117:110–7. doi: 10.1111/BJU.12940, PMID: 25252180

[28] Ivanyi P Beutel G Drewes N Pirr J Kielstein JT Morgan M. Therapy of treatment-related hypertension in metastatic renal-cell cancer patients receiving sunitinib. Clin Genitourin Cancer. (2017) 15:280–290.e3. doi: 10.1016/J.CLGC.2016.10.004, PMID: 27863831

[29] Kumar R Crouthamel MC Rominger DH Gontarek RR Tummino PJ Levin RA. Myelosuppression and kinase selectivity of multikinase angiogenesis inhibitors. Br J Cancer. (2009) 101(10):1717–23. doi: 10.1038/sj.bjc.6605366, PMID: 19844230 PMC2768111

[30] Barber NA Afzal W Akhtari M. Hematologic toxicities of small molecule tyrosine kinase inhibitors. Target Oncol. (2011) 6:203–15. doi: 10.1007/s11523-011-0202-9, PMID: 22127751

[31] Daher-Reyes GS Bence-Bruckler I Busque L Forrest DL Savoie L Keating M-M. Comprehensive analysis of hematological parameter changes after TKI discontinuation for treatment-free remission attempt. Blood. (2019) 134:1653. doi: 10.1182/blood-2019-131599

[32] Fachi MM Tonin FS Leonart LP Rotta I Fernandez-Llimos F Pontarolo R. Hematological adverse events associated with tyrosine kinase inhibitors in chronic myeloid leukemia: A network meta-analysis. Br J Clin Pharmacol. (2019) 85(10):2280–2291. doi: 10.1111/bcp.13933, PMID: 30907446 PMC6783623

[33] Hong MH Kim HS Kim C Ahn JR Chon HJ Shin S-J. Treatment outcomes of sunitinib treatment in advanced renal cell carcinoma patients: A single cancer center experience in Korea. Cancer Res Treat. (2009) 41(2):67–72. doi: 10.4143/crt.2009.41.2.67, PMID: 19707503 PMC2731208

[34] Porta C Gore ME Rini BI Escudier B Hariharan S Charles LP. Long-term safety of sunitinib in metastatic renal cell carcinoma. Eur Urol. (2016) 69:345–51. doi: 10.1016/J.EURURO.2015.07.006, PMID: 26215605 PMC5032140

[35] Arena C Troiano G De Lillo A Testa NF Lo Muzio L. Stomatitis and VEGFR-tyrosine kinase inhibitors (VR-TKIs): A review of current literature in 4369 patients. BioMed Res Int. (2018) 2018:5035217. doi: 10.1155/2018/5035217, PMID: 29992147 PMC5994328

[36] Wu J Huang H. Acquired hypothyroidism in patients with metastatic renal cell carcinoma treated with tyrosine kinase inhibitors. Drug Des Devel Ther. (2020) 14:3977–82. doi: 10.2147/DDDT.S270210, PMID: 33061302 PMC7532040

[37] Vasileiadis T Chrisofos M Safioleas M Kontzoglou K Papazisis K Sdrolia A. Impact of sunitinib-induced hypothyroidism on survival of patients with metastatic renal cancer. BMC Cancer. (2019) 19 (1):407. doi: 10.1186/S12885-019-5610-8, PMID: 31039771 PMC6492389

[38] Badran A Elshenawy MA Shahin A Aljubran A Alzahrani A Eldali A. Efficacy and prognostic factors of sunitinib as first-line therapy for patients with metastatic renal cell carcinoma in an arab population. JCO Glob Oncol. (2020) 6:19–26. doi: 10.1200/JGO.19.00111, PMID: 32031432 PMC6998020

[39] Bozkurt O Karaca H Hacıbekiroglu I Kaplan MA Duzkopru Y Uysal M. Is sunitinib-induced hypothyroidism a predictive clinical marker for better response in metastatic renal cell carcinoma patients? J Chemother. (2016) 28:230–4. doi: 10.1179/1973947815Y.0000000039, PMID: 25948423

[40] Tassi R Baldazzi V Lapini A Carini M Mazzanti R. Hyperlipidemia and hypothyroidism among metastatic renal cell carcinoma patients taking sunitinib malate. Related or unrelated adverse events? Clin Genitourin Cancer. (2015) 13:e101–5. doi: 10.1016/J.CLGC.2014.08.009, PMID: 25450040

[41] Lee WJ Lee JL Chang SE Lee MW Kang YK Choi JH. Cutaneous adverse effects in patients treated with the multitargeted kinase inhibitors sorafenib and sunitinib. Br J Dermatol. (2009) 161:1045–51. doi: 10.1111/J.1365-2133.2009.09290.X, PMID: 19558553

[42] Shyam Sunder S Sharma UC Pokharel S. Adverse effects of tyrosine kinase inhibitors in cancer therapy: pathophysiology, mechanisms and clinical management. Signal Transduct Target Ther. (2023) 8(1):262. doi: 10.1038/s41392-023-01469-6, PMID: 37414756 PMC10326056

[43] Xiong Y Wang Q Liu Y Wei J Chen X. Renal adverse reactions of tyrosine kinase inhibitors in the treatment of tumors: A Bayesian network meta-analysis. Front Pharmacol. (2022) 13:1023660. doi: 10.3389/fphar.2022.1023660, PMID: 36408227 PMC9669664

[44] Ren S Chen X Zheng Y Chen T Hu X Feng Y. Adverse renal outcomes following targeted therapies in renal cell carcinoma: a systematic review and meta-analysis. Front Pharmacol. (2024) 15:1409022. doi: 10.3389/fphar.2024.1409022, PMID: 38989147 PMC11234087

[45] Macfarlane R Heng DYC Xie W Knox JJ McDermott DF Rini BI. The impact of kidney function on the outcome of metastatic renal cell carcinoma patients treated with vascular endothelial growth factor-targeted therapy. Cancer. (2012) 118(2):365–70. doi: 10.1002/cncr.26201, PMID: 21717427

[46] Kato T Kurasawa S Takezawa K Fujiwara Y Yasuda Y Ando Y. Efficacy and safety of anti-angiogenic agents for cancer patients with proteinuria or a history of proteinuria: A systematic review. Anticancer Res. (2024) 44(3):889–894. doi: 10.21873/anticanres.16882, PMID: 38423640

[47] Mielczarek Ł Brodziak A Sobczuk P Kawecki M Cudnoch-Jędrzejewska A Czarnecka AM. Renal toxicity of targeted therapies for renal cell carcinoma in patients with normal and impaired kidney function. Cancer Chemother Pharmacol. (2021) 87:723–42. doi: 10.1007/s00280-021-04260-y, PMID: 33768301 PMC8110505

[48] Gupta S Parsa V Heilbrun LKb Smith DW Dickow B Heath E. Safety and efficacy of molecularly targeted agents in patients with metastatic kidney cancer with renal dysfunction. Anti-Cancer Drugs. (2011) 22(8):794–800. doi: 10.1097/CAD.0b013e328346af0d, PMID: 21799472 PMC3149855

[49] Takahashi S. Fatigue and its management in cancer patients undergoing VEGFR-TKI therapy. Expert Opin Drug Saf. (2022) 21(3):397–406. doi: 10.1080/14740338.2021.1969360, PMID: 34461788

[50] Available online at: https://www.nccn.org/professionals/physician_gls/pdf/fatigue.pdf (Accessed June 29, 2025).

[51] Anand D Escalante CP. Ongoing screening and treatment to potentially reduce tyrosine kinase inhibitor-related fatigue in renal cell carcinoma. J Pain Symptom Manage. (2015) 50(1):108–17. doi: 10.1016/j.jpainsymman.2015.02.007, PMID: 25701692

[52] Santoni M Conti A Massari F Arnaldi G Iacovelli R Rizzo M. Treatment-related fatigue with sorafenib, sunitinib and pazopanib in patients with advanced solid tumors: An up-to-date review and meta-analysis of clinical trials. Int J Cancer. (2015) 136(1):1–10. doi: 10.1002/ijc.28715, PMID: 24415642

[53] Ekenel M Karabulut S Cil I Zırtıloglu A Aydın E Tural D. Sunitinib versus pazopanib for patients with metastatic renal cell carcinoma: 2 Turkish hospital experience. Actas Urol Esp (Engl Ed). (2020) 44:27–33. doi: 10.1016/j.acuro.2019.06.007, PMID: 31744648

[54] Motzer RJ Tannir NM McDermott DF Arén Frontera O Melichar B Choueiri TK. Nivolumab plus Ipilimumab versus Sunitinib in Advanced Renal-Cell Carcinoma. N Engl J Med. (2018) 378:1277–90. doi: 10.1056/NEJMoa1712126, PMID: 29562145 PMC5972549

[55] Motzer R Alekseev B Rha SY Porta C Eto M Powles T. Lenvatinib plus pembrolizumab or everolimus for advanced renal cell carcinoma. N Engl J Med. (2021) 384:1289–300. doi: 10.1056/NEJMoa2035716, PMID: 33616314

[56] Choueiri TK Powles T Burotto M Escudier B Bourlon MT Zurawski B. Nivolumab plus Cabozantinib versus Sunitinib for Advanced Renal-Cell Carcinoma. N Engl J Med. (2021) 384:829–41. doi: 10.1056/NEJMoa2026982, PMID: 33657295 PMC8436591

[57] Rini BI Plimack ER Stus V Gafanov R Hawkins R Nosov D. Pembrolizumab plus Axitinib versus Sunitinib for Advanced Renal-Cell Carcinoma. N Engl J Med. (2019) 380:1116–27. doi: 10.1056/NEJMoa1816714, PMID: 30779529

[58] Claudiani S Apperley JF Szydlo R Khan A Nesr G Hayden C. TKI dose reduction can effectively maintain major molecular remission in patients with chronic myeloid leukemia. Brit J Hematol. (2021) 193(2):346–55. doi: 10.1111/bjh.17286, PMID: 33368155

[59] Powles T Sarwar N Jones R Wilson P Boleti E Protheroe A. An indirect comparison of the toxicity of sunitinib and pazopanib in metastatic clear cell renal cancer. Eur J Cancer. (2012) 48:3171–6. doi: 10.1016/j.ejca.2012.05.022, PMID: 22766517

[60] Atkinson BJ Kalra S Wang X Bathala T Corn P Tannir NM. Clinical outcomes for patients with metastatic renal cell carcinoma treated with alternative sunitinib schedules. J Urol. (2014) 191:611–8. doi: 10.1016/j.juro.2013.08.090, PMID: 24018239 PMC4015627

[61] Mouillet G Paillard MJ Maurina T Vernerey D Nguyen Tan Hon T Almotlak H. Open-label, randomized multicenter phase II study to assess the efficacy and tolerability of sunitinib by dose administration regimen (dose modification or dose interruptions) in patients with advanced or metastatic renal cell carcinoma: study protocol of the SURF trial. Trials. (2018) 19:221. doi: 10.1186/s13063-018-2613-8, PMID: 29650037 PMC5898055

[62] Ito T Yamamoto K Furukawa J Harada K Fujisawa M Omura T. Association of sunitinib concentration and clinical outcome in patients with metastatic renal cell carcinoma treated with a 2-week-on and 1-week-off schedule. J Clin Pharm Ther. (2022) 47:81–8. doi: 10.1111/jcpt.13517, PMID: 34669974

